# Brief psychotic disorder related to areca nut use: a case report

**DOI:** 10.3389/fpsyt.2024.1360493

**Published:** 2024-05-13

**Authors:** Sixun Li, Zhixiong Li, Juan Chen, Feng Wang, Ying Ou, Yi Huang, Zhe Li

**Affiliations:** ^1^ Mental Health Center, West China Hospital, Sichuan University, Chengdu, Sichuan, China; ^2^ Sichuan Clinical Medical Research Center for Mental Disorders, Chengdu, Sichuan, China; ^3^ The Third Department of Clinical Psychology, Karamay Municipal People’s Hospital, Karamay, Xinjiang, China

**Keywords:** brief psychotic disorder, areca nut, betel nut, arecoline, case report

## Abstract

**Background:**

Areca Nut (AN) is the fourth most commonly abused drug after nicotine, ethanol, and caffeine, due to its psychoactive properties provided by bioactive substances. Although previous studies have demonstrated AN’s anxiolytic-like activity and potential benefits in ameliorating symptoms of depression and schizophrenia, there remains limited awareness regarding its association with brief psychotic disorder.

**Case presentation:**

This case report presents the clinical profile of a 30-year-old male patient with a history of betel nut chewing for the past 2 years, who exhibited sudden onset delusions, hallucinations, and disorganized speech and behavior upon increasing the dosage of betel nut consumption. The patient displayed a positive response to antipsychotic treatment, and symptoms resolved upon discontinuation of betel nut consumption. However, one month after discharge, the patient experienced a recurrence of auditory hallucinations upon resuming betel nut chewing. Through counseling and support, the importance of abstaining from betel nut use and maintaining medication compliance was emphasized, resulting in no recurrence of psychotic symptoms during the six-month follow-up.

**Conclusions:**

This case report highlights the related role of betel nut with brief psychotic disorder, especially when the chewing dosage is abruptly increased. It underscores the importance of considering betel nut as a potential precipitant related to acute psychiatric disorders in clinical settings.

## Background

Areca nut (AN) is the fruit of the palm tree Areca cattechu Linn. AN chewers are estimated to number more than 600 million people, primarily located in Asian countries such as India, Pakistan and Taiwan as well as among migrants from South Africa and the UK, constituting account at least 10% of the world population (1). A survey conducted in the United States indicated that 17% of respondents self-reported having used AN products, while 31% reported having friends or family members who were AN users (2). In China, there are approximately 60 million chewers (3). On the one hand, AN has been utilized as a traditional herbal remedy for a long time. Its’ pharmacological effects encompass a wide range of purported benefits, including its potential to alleviate gastric and intestinal diseases, depression, anxiety, liver diseases, nerve diseases, bacterial infections, and promote skin protection (4). AN has also been attributed with anthelmintic efficacy, antioxidant properties, and anti-hypoxia and anti-osteoarthritis effects (5). On the other hand, AN holds cultural and traditional significance in several countries. In these areas, people have been chewing betel nut since ancient times, and this custom has been prosperous. Betel nut has even been given deeper social significance, being considered a necessary product for weddings or religious sacrifices (6).

At present, areca nut has been regarded as the fourth most commonly abused drug after nicotine, ethanol, and caffeine (7), owing to its psychoactive properties (6). Users report experiencing pleasurable and stimulating effects, including a sense of well-being, euphoria, heightened alertness, a warm bodily sensation, and increased work capacity (8). With the widespread use of betel nut, more attention has been paid to its health risks. In fact, areca nut has been classified as carcinogenic to humans and has been linked to a range of detrimental health effects, including carcinogenicity, genetic and epigenetic instability, progression of sub-mucous fibrosis, oral cancer, and liver disease (5).

Regarding the relationship between betel nut consumption and psychotic disorder, although instances of psychosis induced by areca nut consumption were first documented in 1977 in Papua New Guinea, such cases have been infrequently reported in the literature since (9). In this context, we present a case report detailing a brief psychotic disorder related to areca nut chewing, underscoring the potential psychiatric implications associated with this widely used substance. This report serves as a reminder to healthcare professionals of the possible risks linked to areca nut consumption, necessitating increased vigilance in educating the public about its potential adverse effects on mental health.

## Case presentation

The subject of this case study is a 30-year-old married male, who was admitted to our hospital on May 9, 2023, displaying symptoms of delusions, hallucinations, disorganized speech, and aberrant behavior that had persisted for a duration of 7 days. Notably, seven days prior to admission, the patient exhibited hostile behavior, verbally confronting an external entity he perceived as cursing at him through his window. Additionally, he expressed fears of being targeted for harm, resulting in heightened anxiety and social withdrawal. Furthermore, the patient engaged in repetitive questioning regarding potential theft of his finances, causing significant distress to himself and his family. Approximately four days before hospitalization, the patient’s symptoms worsened, leading to insomnia, during which he incessantly initiated conversations with his wife, despite their incoherence. Moreover, the patient began engaging in self-directed speech with incomprehensible content, further complicating the situation. Concerned by his escalating behavior, the patient accompanied his wife and child to the police station to report the perceived incidents. Alarmingly, during sleep, he resorted to placing a knife under his pillow, a behavior he deemed necessary to attain a sense of security. Two days before admission, the patient’s nocturnal activities became even more concerning, as his family observed him engaging in dance-like movements and forcefully pounding the bed while still holding a knife. Though conscious and communicative, the patient struggled to offer a rational explanation for his unusual conduct.

As for the history of past illness, there were no reports of significant past illnesses or medical conditions. The patient also had no past history of psychiatric illnesses. Moreover, both the patient and his family members denied any history of smoking, alcohol consumption, or the use of psychoactive substances. Concerning personal history, during childhood, the patient achieved developmental milestones within the expected timeframe. His academic performance was average, and he pursued physical education at a sports school during junior high school. Notably, for the past 2 years, the patient had been a habitual chewer of betel nuts, consuming approximately 10 nuts daily. Upon discontinuation of betel nut consumption, he experienced mild irritability and restlessness, suggesting a possible withdrawal effect. However, seven days before admission, the patient escalate his betel nut intake to approximately 15-20 nuts per day. Regarding family history, no significant findings were reported in the patient’s family history, indicating an absence of notable psychiatric or medical conditions among immediate family members.

### Physical, mental and laboratory examinations

Upon examination, the patient’s vital signs were stable, and cardiovascular, respiratory, abdominal, and neurological assessments yielded unremarkable results. In the mental examination, the patient demonstrated a voluntary admission, appropriate attire, a natural facial expression, and adequate self-care capabilities. He displayed good attention and provided relevant responses during conversations, albeit occasionally moving around. Despite showing good orientation, the patient lacked insight into his condition. A rough assessment of cognitive function revealed normal intelligence, memory, and calculation abilities. Notably, he reported experiencing auditory hallucinations, feelings of persecution, and excessive suspicion. Regarding his emotional state, there were no indications of depressive syndrome, anxiety syndrome, or manic syndrome. Will and behavior did not show evidence of pathologically increased or decreased volition, but a disrupted sleep-wake rhythm was noted. The results of routine blood, urine, and stool tests were within normal limits, as were blood glucose levels and liver and renal functions, with no signs of infection. Thyroid function tests yielded normal results. Notably, DIC profile, BNP, and cardiac markers presented no abnormalities. Tests related to anemia and malignancy yielded unremarkable findings. Pre-transfusion tests and toxicological screening were normal, and blood ethanol concentration was found to be below 10mg/dl (reference range, <10mg/dl). Electrocardiogram, electroencephalogram, echocardiography, thyroid ultrasound, chest CT, head MRI, abdominal ultrasound, and male urogenital ultrasound showed no significant abnormalities. Serum level measurements were conducted, revealing the following results: IL-1β at 7 pg/mL (reference range, 0-5 pg/mL), soluble interleukin-2 receptor (sIL-2R) at 750 U/mL (reference range, 223-710 U/mL), IL-6 at 9 pg/mL (reference range, 0-7 pg/mL), IL-8 at 49 pg/mL (reference range, 0-62 pg/mL), IL-10 at 8.6 pg/mL (reference range, 0-9.1 pg/mL), tumor necrosis factor-α (TNF-α) at 7.7 pg/mL (reference range, <8.1 pg/mL), and CRP at 5.5 mg/L (reference range, <5 mg/L).

### Further diagnostic work-up

The patient underwent an extensive psychiatric assessment administered by a seasoned psychiatrist. The evaluation incorporated the Chinese versions of two standardized scales: the Hamilton Anxiety Rating Scale (HAMA) and the 24-item Hamilton Depression Rating Scale (HAMD). The HAMA yielded a score of 7, denoting the absence of anxiety symptoms, while the HAMD resulted in a score of 8, indicating an absence of depressive symptoms. Moreover, the Brief Psychiatric Rating Scale (BPRS) was employed, with the patient scoring 64. Notably, BPRS total scores typically fall within a range of 18 to 126, with higher scores indicative of more severe symptoms. The patient’s BPRS score of 64 signifies the presence of significant psychotic symptoms. The Naranjo Causality Scale (adapted) (10) was also employed to explicitly establish the relationship between AN and the symptoms of this patient, resulting in a score of 9, indicating a definite relationship between the use of AN and the appearance of the symptoms.

### Final diagnosis

The patient’s clinical presentation and history align with the diagnostic criteria outlined in the 5th edition of the Diagnostic and Statistical Manual of Mental Disorders (DSM-5) for brief psychotic disorder. This diagnosis is characterized by the sudden onset of psychotic symptoms, such as delusions, hallucinations, disorganized speech, or grossly disorganized or catatonic behavior, lasting for a brief period and eventually resolving within one month.

### Treatment and outcome

Following admission, the patient promptly discontinued betel nut consumption and commenced treatment with sustained-release paliperidone at a daily dose of 3 mg and olanzapine at 5 mg for antipsychotic management and improved sleep therapy at night. Subsequently, over the course of 5 days in the hospital, the patient’s psychiatric symptoms exhibited a gradual amelioration. Hallucinations, persecutory feelings, and other psychotic manifestations ceased to occur. Furthermore, the aberrant speech and behavior, including self-directed conversations and nocturnal dancing on the bed, did not reoccur. Additionally, the patient’s excessive suspicion demonstrated marked improvement compared to the state prior to hospitalization. Remarkably, after a total of 10 days of hospitalization, the patient showed substantial progress, and successful discharge was achieved, with a notably reduced BPRS score of 19, indicating significant improvement in his overall psychiatric symptoms. A follow-up analysis of the patient’s serum cytokine levels revealed notable changes. sIL-2R levels were observed at 700 U/mL (reference range, 223-710 U/mL). Moreover, IL-8 measured 38 pg/mL (reference range, 0-62 pg/mL), and IL-10 measured 7.1 pg/mL (reference range, 0-9.1 pg/mL), both are within normal limits. The levels of IL-1β were measured at 6 pg/mL (reference range, 0-5 pg/mL), while IL-6 was recorded at 8 pg/mL (reference range, 0-7 pg/mL), both indicating near-normal values. Similarly, the tumor necrosis factor-α (TNF-α) level measured 6.9 pg/mL (reference range, 8.1 pg/mL), and C-reactive protein (CRP) level measured 4.5 mg/L (reference range, <5 mg/L), both indicating a return to within the reference range.

### Follow-up

After discharge, the patient’s family members supervised his medication regimen, ensuring he continued his prescribed treatment without interruption. One-month post- discharge, the patient attended a gathering where he consumed 7 betel nuts, leading to a recurrence of auditory hallucinations, with the patient perceiving someone urging him to engage in physical altercation. Promptly, his family brought him to our outpatient clinic for assessment. The patient’s psychiatric status was evaluated using the BPRS, yielding a score of 42, indicative a significant worsening of his psychotic symptoms compared to the score upon discharge. Although the patient’s serum cytokine levels of IL-8 (60 pg/mL), IL-10 (8.1 pg/mL), TNF-α (8.0 pg/mL), and CRP (4.8 mg/L) were within the normal range, there were notable increases in IL-1β (10 pg/mL), sIL-2R (790 U/mL), and IL-6 (9 pg/mL) compared to the previous measurements. Emphasizing the significance of abstaining from betel nut consumption, the patient received comprehensive counseling and support. The ongoing medication treatment, comprising 3mg sustained-release paliperidone and 5mg olanzapine, was continued, and the patient was advised to attend regular follow-up visits at the outpatient clinic to closely monitor his condition. Over the course of the subsequent half-year follow-up period, the patient adhered to the advice and refrained from further consumption of betel nuts. Encouragingly, he did not report any recurrence of similar psychotic symptoms during this period, as evidenced by a BPRS score of 18, indicative of notable improvement in his psychiatric status compared to previous assessments. Monitoring of the patient’s serum cytokine levels during the follow-up period showed a trend towards normalization. Specifically, the levels of IL-1β decreased to 4 pg/mL, sIL-2R reduced to 610 U/mL, IL-6 dropped to 5 pg/mL, IL-8 decreased to 40 pg/mL, IL-10 reduced to 6.1 pg/mL, TNF-α reduced to 6.9 pg/mL, and CRP increased slightly to 5.0 mg/L.

## Discussion and conclusions

Common causes of brief psychotic disorder include: (1) Drug-induced Psychosis: Certain drugs, such as hallucinogens (e.g., LSD, magic mushrooms), stimulants (e.g., cocaine, amphetamines), alcohol, and some prescription medications (e.g., benzodiazepines), can trigger transient psychotic symptoms. (2) Substance Intoxication: Heavy metal poisoning (e.g., lead, mercury) and organic solvent poisoning can lead to temporary psychosis when individuals are exposed to toxic substances. (3) Medical Conditions and Infections: High fever, central nervous system infections, meningitis, and autoimmune encephalitis are among the medical conditions that can provoke brief psychotic episodes. (4) Acute Stress Reactions: Severe trauma, violent events, disasters, or other extreme stress situations can induce a transient psychotic response in susceptible individuals (11).

In this particular case, the patient had a history of prolonged betel nut consumption, and notably, there was a significant escalation in betel nut chewing behavior prior to the emergence of symptoms. The disease exhibited a rapid progression, primarily manifesting as a psychotic disorder characterized by hallucinations and delusions. The duration of the illness was short-lived, and following one week of abstaining from betel nut consumption and receiving paliperidone and olanzapine for symptomatic treatment, the patient’s symptoms displayed considerable improvement. However, one month after discharge, a relapse occurred when the patient resumed betel nut chewing, resulting in the recurrence of auditory hallucinations. In response, counseling and support were promptly provided to underscore the critical importance of refraining from betel nut usage while maintaining the prescribed medication regimen. Subsequent half-year follow-ups revealed no recurrence of psychotic symptoms, indicating the effectiveness of the intervention.

The patient’s medical history, urine toxicology, and blood ethanol screening upon admission all showed no evidence of exposure to or use of other psychoactive substances. Additionally, there were no indications of recent changes in the patient’s living or working environment, and he denied any exposure to heavy metals, paints, or organic solvents. The absence of fever prior to or during the course of the illness and the unremarkable neurological examination further ruled out other potential medical or infectious causes. While the patient did experience certain life events recently, they did not appear to be traumatic events by most people’s perception. Given the lack of any other identifiable triggers, the relationship between the patient’s psychotic disorder and betel nut chewing cannot be overlooked. Taking into account all the available information, the final diagnosis in this case is brief psychotic disorder related to Areca nut use. The significant increase in betel nut consumption prior to the onset of symptoms, the rapid progression of the illness, and the subsequent improvement upon discontinuing betel nut chewing and receiving appropriate antipsychotic treatment, furthermore, the recurrence of symptoms occurred when the patient resumed the use of betel nut. All strongly support the role of betel nut in inducing the psychotic symptoms observed in the patient.

As an ancient Chinese herb with a long history, the traditional usage of Areca nut (AN) was applied for treating several diseases including malaria, diarrhea, ascariasis, edema, stagnation of food, arthritis and beriberi. In recent years, some studies have investigated the impact of AN on individuals with schizophrenia. A cross-sectional study involving 70 participants with schizophrenia found that betel chewers scored significantly lower on the positive and negative sub-scales of the Positive and Negative Syndrome Scale (PANSS) compared to non-chewers (12). However, a subsequent longitudinal study could only replicate these findings in male high-consumption chewers, specifically in the positive symptoms cluster of the PANSS (13). In contrast, another longitudinal study failed to demonstrate any significant difference in symptoms between betel users and non-users (14). Overall, the collective findings from the literature suggest that betel nut consumption may have potential therapeutic effects on schizophrenia symptoms in some cases, mainly considering the potential of arecoline to modulate neurotransmitter systems implicated in schizophrenia. In this particular case, the patient’s prolonged betel nut consumption and the subsequent onset of psychotic symptoms after increasing dosage, followed by symptom resolution upon discontinuation, and recurrence after resuming betel nut use, provide compelling observations suggesting that the relationship between the psychotic symptoms cannot be ignored in clinical practice. These findings underscore the need for further research to comprehensively understand the relationship between betel nut and psychotic symptoms.

According to the results of previous literature search, there were almost no reports of betel nut related psychotic disorder, and this case report even seems to contradict previous findings that arecoline can improve psychotic symptoms. The possible reason for the scarcity of reports on psychotic disorder related to betel nut may be as follows: the diagnosis of mental diseases often relies on medical history rather than objective biological markers, and betel nut is often regarded as a food rather than a drug. Medical staff often pay little attention to the use of betel nut in the consultation process of patients with acute and transient mental disorders, and patients and their families rarely mention it actively. The reason why this case report seems to contradict previous research results that arecoline can improve psychotic symptoms may be related to arecoline, the main active substance of AN, which has a variety of psychoactive effects. On the one hand, arecoline can reduced the dopaminergic hyperactivity through the modulation of M1, M2 and M4 receptors to ameliorate the negative symptoms of psychosis (15). On the other hand, arecoline exhibits monoamine oxidase-A (MAO-A) inhibitor-like properties, preventing the breakdown of neurotransmitters such as dopamine, norepinephrine, and serotonin in the brain (16). This action leads to increased concentrations of these neurotransmitters, which are essential in regulating mood and behavior. Meanwhile, arecaidine, the main metabolite of arecoline, acts as a competitive inhibitor of γ-aminobutyric acid (GABA) (17, 18). GABA is the primary inhibitory neurotransmitter in the brain and plays a crucial role in modulating neuronal activity. In our presented case, the psychotic symptoms may be linked to the modulation of dopamine hyperactivity by arecoline and the inhibitory effects on GABA (19), particularly due to the sudden increase in betel nut dosage. Furthermore, studies have suggested that betel nut use may trigger inflammatory reactions (20) and activate the immune system (21). While the exact etiology of psychotic disorders remains unclear, the production of various inflammatory cytokines and free radicals by activated microglia may contribute to neurotoxic effects in these conditions (22, 23). Serum levels of inflammatory cytokines were elevated in our patient upon admission, and they returned to normal after ceasing betel nut consumption and receiving antipsychotic treatment. Intriguingly, the serum levels of inflammatory cytokines increased again after resuming betel nut use. Monitoring of the patient’s serum cytokine levels during the follow-up period showed a trend towards normalization. This suggests that the psychotic symptoms in our patient may be related to abnormal inflammatory factor release. Nevertheless, a comprehensive understanding of the specific etiological relationship between betel nut and inflammatory reactions requires further investigation.

In conclusion, this case report provides a significant example of brief psychotic disorder related to betel nut chewing, particularly in the context of a sudden increase in dosage. Although the results reported in this case differ from evidence obtained from previous systematic studies and a prospective study with relatively large samples, and considering that the association with betel nut could be incidental, we find it difficult to make a definitive conclusion regarding whether betel nut leads to mental disorders. However, this case report still provides some insights for the diagnosis and treatment of patients with mental disorders in clinical practice. In clinical practice, it is imperative to thoroughly inquire about the patient’s history of betel nut consumption when encountering individuals with brief psychotic disorder. Furthermore, the dynamic monitoring of serum levels of inflammatory cytokines may hold importance for patients who consume betel nut. Considering the potential link between betel nut use and inflammatory reactions, monitoring inflammatory markers can aid in assessing the impact of betel nut consumption on the patient’s health and guiding treatment decisions. Importantly, arecoline, as a major component of betel nut, exhibits a range of pharmacological effects. However, it is crucial to recognize that arecoline may also have potential side effects. As seen in this case, betel nut consumption can lead to the development of psychotic symptoms, indicating the need for cautious consumption practices and appropriate medical supervision.

## Data availability statement

The original contributions presented in the study are included in the article/supplementary material. Further inquiries can be directed to the corresponding author.

## Ethics statement

The studies involving humans were approved by West China Hospital Ethics Committee. The studies were conducted in accordance with the local legislation and institutional requirements. The participants provided their written informed consent to participate in this study. Written informed consent was obtained from the individual(s) for the publication of any potentially identifiable images or data included in this article.

## Author contributions

SL: Writing – original draft, Writing – review & editing. ZXL: Writing – review & editing. JC: Writing – review & editing, Methodology, Project administration, Investigation. FW: Writing – review & editing, Supervision, Project administration, Investigation. YO: Writing – review & editing. YH: Writing – review & editing. ZL: Funding acquisition, Writing – review & editing. 
